# Bio-fabrication of Zinc Oxide nanoparticles to rescue Mung Bean against Cercospora leaf spot disease

**DOI:** 10.3389/fpls.2022.1052984

**Published:** 2022-11-29

**Authors:** Hamza Rafiq, Zill-e-Huma Aftab, Tehmina Anjum, Basharat Ali, Waheed Akram, Uzma Bashir, Faisal Shafiq Mirza, Muzammil Aftab, Muhammad Danish Ali, Guihua Li

**Affiliations:** ^1^ Department of Plant Pathology, Faculty of Agricultural Sciences, University of the Punjab, Lahore, Pakistan; ^2^ Guangdong Key Laboratory for New Technology Research of Vegetables/Vegetable Research Institute, Guangdong Academy of Agricultural Sciences, Guangzhou, China; ^3^ Department of Physics, Government College University, Lahore, Pakistan; ^4^ Institute of Microbiology and Molecular Genetics, University of the Punjab, Lahore, Pakistan; ^5^ Department of Science and Humanities, National University of Computer and Emerging Sciences- FAST, Lahore, Pakistan; ^6^ Department of Physics, University of the Punjab, Lahore, Pakistan

**Keywords:** zinc oxide nanoparticles, green synthesis, trachyspermum ammi, cercospora leaf spot, antifungal

## Abstract

Plant disease management using nanotechnology is evolving continuously across the world. The purpose of this study was to determine the effect of different concentrations of green synthesized zinc oxide nanoparticles (ZnO NPs) using *Trachyspermum ammi* seed extract on Cercospora leaf spot disease in mung bean plants under *in-vitro* and *in-planta* conditions. Additionally, the effects on mung bean agronomic and physiological parameters were also assessed. The green synthesized ZnO NPs were characterized using UV-visible spectroscopy, Fourier-transform infrared spectroscopy (FTIR), X-ray diffraction (XRD) and Scanning electron microscopy (SEM). Green synthesized NPs were tested for their ability to inhibit fungal growth at five different concentrations under *in-vitro* experiment. After 7 days of inoculation, ZnO NPs (1200 ppm) inhibited mycelial growth substantially (89.86% ± 0.70). The *in-planta* experiment showed statistically significant result of disease control (30% ± 11.54) in response to 1200 ppm ZnO NPs. The same treatment showed statistically significant improvements in shoot length, root length, number of leaves, number of pods, shoot fresh weight (28.62%), shoot dry weight (85.18%), root fresh weight (38.88%), and root dry weight (38.88%) compared to the control. Our findings show that green synthesized ZnO NPs can control *Cercospora canescens* in mung bean, pointing to their use in plant disease control and growth enhancement.

## Introduction

Over the past few decades, phytopathogens causing microbial infections have become a serious challenge for humans because the agrochemicals used for their control yield no result as the microbes have upgraded themselves to superbugs by developing resistance against commonly used pesticides. World Health Organization (WHO) have pointed out various suggestions for antimicrobial resistance, initiating awareness after obtaining true knowledge about microbes and their antimicrobial resistance, trying to reduce the infection rate by optimizing the exact amount of antibiotics against a specific pathogen, and mainly supported by funds to do research on antimicrobial resistance and treat the diseases (WHO, 2021). Conventionally it was a well-accepted fact that several control measures like field sanitation, application of proper fertilizers, etc. can help in controlling the spread of diseases but now at some stage, these measures become ineffective in eradicating the diseases properly. Therefore, it is necessary to develop smart, efficient, and effective materials which provide good candidature for antimicrobial agents (Fincheira et al., 2021). This problem can be solved by nanotechnology by providing pesticides in the form of nanoparticles, nanoemulsions, and nanoencapsulation, which can provide an eco-friendly cost-effective solution for the plant disease management or crop protection sector (Panpatte et al., 2016; Ul Haq and Ijaz, 2019). The use of nanomaterials in agriculture ensures the nominal use of agrochemicals ultimately reducing soil pollution and conserving the soil microbial communities (Shang et al., 2019; Ul Haq and Ijaz, 2019; Bahrulolum et al., 2021). Recently Kumar et al. (2020) found that nanorods of pure CdO and Zn-doped CdO can be utilized as good antibacterial agents in the pharmaceutical and biomedical sectors. Likewise, Aftab et al. (2021; 2022); Aftab et al. (2020) have reported that Cu-doped NiO thin films convey incredibly good anti-fungal as well as anti-bacterial characteristics. Furthermore, there are numerous other reports present in the literature in which other metals nanoparticles and their oxides have been used as antimicrobial agents (Aljabali et al., 2018; Roy et al., 2019; Adewale et al., 2020; Chand et al., 2020; Pachaiappan et al., 2021).

Among these oxides, zinc oxide (ZnO) is one of the most commonly used materials due to its large number of applications like it is used in food packaging materials offering preservatory effects (Espitia et al., 2012). Its nanoparticles are used in paints, sunscreens, and coatings owing to their transparency to visible light and high absorption in the ultra-violet regions (Franklin et al., 2007). Moreover, they are used as an antibacterial ingredient in ointments, creams, lotions, ceramics, self-cleaning glasses, and deodorants (Jamdagni et al., 2018). Ansari et al. (2012) assessed the antimicrobial potential of ZnO nanoparticles and reported that they seem to possess both antifungal and antibacterial properties. Both Gram-negative and Gram-positive bacteria were assessed, and considerable activity was observed against resistant bacterial spores. They found that ZnO nanoparticles when doped with other metals such as silver, gold, chromium, etc. improved the antimicrobial activity (Shah et al., 2014; Jiménez et al., 2015). They also reported that smaller nanoparticles offer better inhibitions in higher concentrations. In another study, Archana et al. (2022) prepared zinc oxide nanoparticles from *Cucumis melo* and examined them by XRD, FTIR, UV-visible Spectroscopy, SEM, and EDX analysis and then used them for their antibacterial activity. The crystallite size was obtained to be 12.8 nm. The zone of inhibition for *E. coli* was observed to be 9.85 ± 0.68 mm suggesting that *Cucumis melo* may act as a good reducing element and may serve as a better antibacterial and antioxidant agent.

Mung bean (*Vigna radiata L.*) also known as “green-gram” is a short-term legume of the family Fabaceae that is enriched with proteins, carbohydrates, and vitamins (Ganesan and Xu, 2018). Globally, mung bean is grown on more than 6 million hectares (approximately 8.5% of the total area under pulses). Annual production of the mung bean crop is around 3 million tons which accounts for nearly 5% of global pulse production (Sehrawat et al., 2019). Mung bean (*Vigna radiata* (L.)) is one of the most important food legume crops and 90% of the global mungbean production came from South, East and Southeast Asia (Nair et al., 2013). In Pakistan, the production of mung bean in 2021-22 increased by 29% compared to last year’s (2020-21) production and is grown on an area of 301800 acres with a production of 263800 tonnes (Anonymous, 2022). Mung bean productivity is constrained by both biotic and abiotic factors. The biotic diseases of mung bean include yellow mosaic, anthracnose, powdery mildew, Cercospora leaf spot (CLS), halo blight, bacterial leaf spot, and tan spot (Nair et al., 2019). Major yield losses are due to the Cercospora leaf spot caused by *Cercospora canescens.* The pathogen affects the foliation and eventually damages whole crop quality that leading to yield loss of up to 50-70% (Chand et al., 2012). Initially, the brownish spots appear on leaves lately on branches, stems, and pods too which ultimately reduce the number/size of pods and seeds.

Fungicides which include Antracol, Ridamil Gold, Mancozeb, Curzate, Champion, Topsin-M, Cabriotop, and Score are being used in commercial agriculture and research and development of novel, effective and target-specific pesticides, at the varying degree of effectiveness to control CLS (Resh and Cardé, 2009; Shahbaz et al., 2014; Abbas et al., 2020). Only 0.1 percent of the used pesticides reach the target pests, while the rest (99.9%) contaminate the environment (Carriger et al., 2006), with major consequences for human health and ultimately the food chain. Due to the harmful aspects of fungicides, easy and environment-friendly alternatives are the best method to control diseases. Other than the currently used systemic fungicides, botanicals are the best-known alternatives which include Neem leaf extract and Kaner leaf extract and have shown 33.29 and 34.03% control respectively (Ram et al., 2018). A plant extract used with micronutrients on the nanoscale depicted more control than micronutrients on the nanoscale and plant extract used separately can enhance this control by up to 80% (Basnet et al., 2018). In the presence of appropriate nanomaterials, slow degradation and controlled release of active components can provide long-term pest control (Chhipa, 2017).

*Trachyspermum ammi L.* (Apiaceae) is commonly famous as Ajwain. In Ayurvedic meds, it is utilized as a restorative plant for its stimulant, carminative, antispasmodic, and tonic properties (Awais Hanif et al., 2021). Due to the presence of various chemical constituents in *T. ammi*, great pharmaceutical and antimicrobial potential have been reported i.e. antifungal, antibacterial, nematicidal, insecticidal, anti-inflammatory, enzyme modulation, antioxidant, and analgesic activity (Chahal et al., 2017; Bashyal and Guha, 2018; Dabhane et al., 2022). *T. ammi* has been used in the green synthesis of gold (Bawazeer et al., 2021; Perveen et al., 2021), zinc (Ali et al., 2022), and magnesium (Dabhane et al., 2022) and has shown to have significant antimicrobial potential under lab conditions.

This study was conducted to provide data on the biosynthesis of ZnO nanoparticles using seed extract of *Trachyspermum ammi* and their antifungal potential of Cercospora leaf spot disease under lab environment and field conditions. To best of our knowledge this planting material was not previously used to synthesize the ZnO NPs and these NPs were not previously assessed against Cercospora leaf spot of mungbean. Alongwith that the effect of ZnO NPs on the agronomic and physiological parameters were also assessed.

## Material & methods

### Collection of plant material

Seeds of *T. ammi* were collected from the local market of Lahore, Pakistan. The mung bean variety (16071) was procured from Ayub Agricultural Research Institute, Faisalabad.

### Isolation and identification of the pathogen

In 2021, the survey was conducted in the mung bean fields at Ayub Agricultural Research Institute (AARI), Faisalabad, Pakistan, for the collection of infected leaf samples showing typical symptoms of Cercospora leaf spot disease. Infected leaf samples were surface sterilized by the standard sodium hypochlorite method (Osdaghi, 2014), and plated onto a 2% Potato Dextrose Agar (PDA) medium containing 10 mg/L of streptomycin. The plates were incubated at 28 ± 2 °C. Fungal colonies were purified by successive sub-culturing onto new media plates. Preliminary identification was performed based on microscopic observation.

Afterward, molecular identification was performed by sequencing the ITS region. Genomic DNA was extracted from the mycelial mass of fungi using the common CTAB method. Internal transcribed spacer (ITS) region was amplified using ITS 1 (5’ -TCCGTAGGTGAACCTGCGG- 3’) and ITS 4 (5’ -TCCTCCGCTTATTGATATGC- 3’) primers as described by (Mohamed et al., 2021). Amplified PCR products were sequenced by Sanger dideoxy sequencing and homology analysis was performed using the BLAST tool.

### Koch’s postulates

Koch’s postulates were applied to each fungus to confirm its pathogenicity. Seven days old cultures of isolated fungi were transferred to potato dextrose broth and kept on a shaker for seven to ten days. A particular spore solution (10^6^ spores/ml) was calibrated using a haemocytometer. Twelve mung bean leaves were washed with sterile distilled water. All these leaves were set in petri dishes with sterile blotting paper on both the lower and upper lids of each plate (separately in six plates). Five microliters of spore suspension were then applied to these leaves, while the remaining six were given five microliters of distilled water (control). The inoculated leaves were kept at 25 °C. Observed disease symptoms were compared with the original symptoms. The pathogen was then re-isolated from these infected leaves, which was then compared to the original isolate of fungus on a 2% PDA medium.

### Green synthesis of zinc oxide nanoparticles

**Figure 1:**
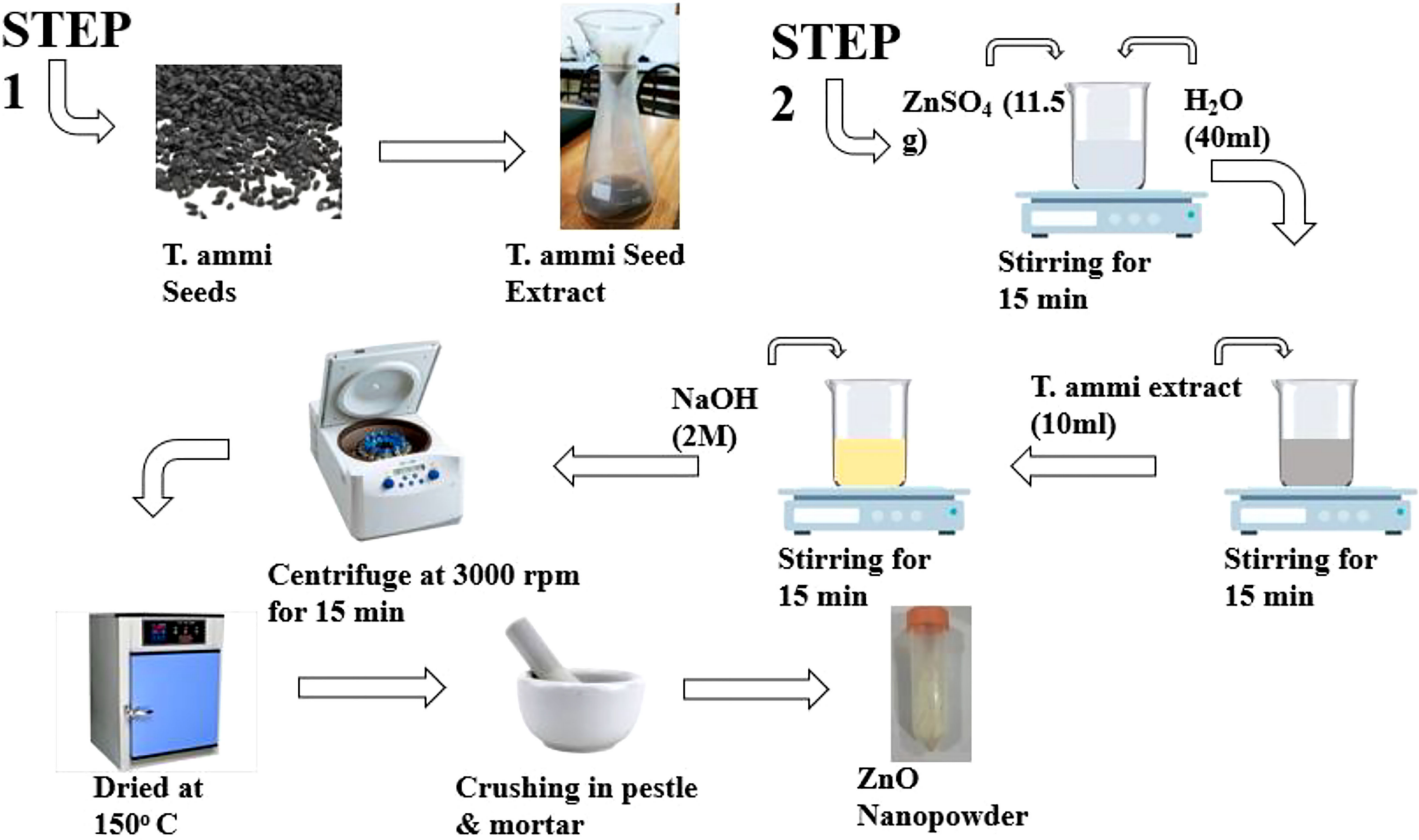
Schematic representation of the green synthesis of ZnO NPs using T. ammi seed extract.

*T. ammi* seeds were ground into a fine powder and the powder (30 g) was boiled for 15 minutes in 150 mL of distilled sterilized water. After cooling the aqueous seed extract, it was filtered using Whatman No. 1 filter paper. The finished seed extract was kept at 4 °C in a sterile container for further use (Pachaiappan et al., 2021). Afterward, zinc sulphate (11.5g) was dissolved in 40 mL of distilled sterilized water and well stirred with a magnetic stirrer. Ten mL of *T. ammi* seed extract was added to this mixture using a sterile injection. The resultant mixture was continually stirred For at least 10 minutes. Sodium hydroxide solution (2 M) was added dropwise to the above solution and stirred for two hours. Finally, the mixture was centrifuged at 3000 rpm for 15 minutes, rinsed several times with distilled sterilized water, and dried in a hot air oven at 150 °C. A fully dried zinc oxide nanopowder was obtained carefully. The whole preparation process of nanoparticles is presented in **Figure 1**. The zinc oxide nanoparticles were transferred to a sterile container for further use characterization (Pachaiappan et al., 2021).

### Characterization of green synthesized zinc oxide nanoparticles

Zinc oxide nanoparticles were characterized using a UV-visible spectrophotometer (Lambda 35, Perkin Elmer, USA) in the wavelength range of 200 to 800 nm. The Attenuated Total Reflection Fourier Transform Infrared (ATR-FTIR) spectra were recorded on a JASCO ATR-FTIR–6600 spectrometer (Japan) fixed with an ATR single reflection diamond for both fingerprint and high wavenumber region ranging from 400–4000 cm^–1^ at a resolution of 4 cm^–1^ and 16 accumulated scans. Morphological characterization was conducted using High-Resolution Scanning Electron Microscopy (HR-SEM).

### Antifungal activity of zinc oxide nanoparticles

The antifungal activity of zinc oxide nanoparticles was analyzed using the agar dilution method (Tryfon et al., 2021). Zinc oxide nanoparticles were sterilized by autoclaving before use. Afterward, nanoparticles were amended in sterilized PDA media at concentrations of 300, 600, 900, 1200, and 1500 µg/mL. The fungi were inoculated in media plates as a disc of 0.5 cm fungal material obtained from a 7-day-old culture. The control treatment was lacking nanoparticles. Antifungal activity was evaluated by measuring the diameter of fungal colonies and the growth inhibition was calculated according to the formula:


$$Inhibition(\%)=\frac{dc-dt}{dc}\times 100$$

Where, dc is the average diameter of linear growth in control, and dt is the average diameter of linear growth in treatment. Five replicates were included in each treatment and the whole experiment was repeated twice.

### *In-planta* antifungal activity of nanoparticles

The experiment was conducted during the zaid rabi, (March-June) season at the Department of Plant Pathology, Faculty of Agricultural Sciences, University of the Punjab, Lahore, Pakistan. Pots of 12-inch diameter were filled with sterilized sandy loam potting media and seeds of *Vigna radiata* of variety (16071) were sown in each pot. Pots were regularly watered. Water suspension of the spores of *Cercospora canesens* was prepared at a concentration of 10^6^ spores per mL. Fifty mL of spore suspension was added to each pot according to the experimental design after 1 week of sowing. The control treatment was provided with 50 mL of sterilized water. Afterward, the aqueous solution of nanoparticles was prepared at different concentrations. Fifty mL of this nanoparticle’s solution was added to every pot on a fortnight basis according to the treatments mentioned in **Table 1**. The whole experiment was performed in triplites and repeated twice.

**Table 1:** Details of treatments.

Sr. No.	Treatment Name	Label
**T_1_**	Negative Control	-ive
**T_2_**	Positive Control (Pathogen)	+ive
**T_3_**	ZnO (900 ppm) + Pathogen	C1+P
**T_4_**	ZnO (1200 ppm) + Pathogen	C2+P
**T_5_**	ZnO (900 ppm)	C1
**T_6_**	ZnO (1200 ppm)	C2

#### Disease parameter

The plant disease index was measured after 60 days of inoculation and was determined using a scale of 0 to 9 (**Supplemental Table 1**). The following formula was used to compute the percentage disease index (PDI).


$$PDI=\frac{Sum\;of\;all\;disease\;severity\;ratings}{Total\;observations\times Highest\;rating\;in\;scale}\times 100$$

#### Growth parameters

Nine different agronomic characters such as root length, shoot length, number of leaves, number of pods, shoot fresh weight, shoot dry weight, root fresh weight, root dry weight, pod size and root nodules were recorded after 75 days post plantation.

#### Estimation of total chlorophyll and carotenoids

For quantification of the total Chl contents, 0.05 g frozen leaf samples were homogenized in 10 ml of 80% acetone using liquid nitrogen (Gong et al., 2022). The mixture was centrifuged to obtain clear supernatant. The OD of supernatant was measured at wavelengths of 663 and 645 nm by a spectrophotometer (UV-2550 Shimadzu, Japan) to evaluate the Chl *a*, *b* and total Chl concentration (mg/L). Carotenoids quantification was performed according to (Alam et al., 2016). Two grams of dried leaf sample was homogenized in 40 mL acetone, 60 mL n-hexane, and 0.1 g MgCO_3_ for 5 min. Vacuum filtration was performed to obtain a clear mixture. Afterward, the filtrate was extracted with 25 mL of acetone and n-hexane. Afterward, the filtrate was further extracted with 100 mL of distilled water. The absorbance value was recorded at 436 nm. Finally, the carotenoid contents were estimated by comparing OD values with the standard curve of β-carotene.

### Statistical analysis

Data were analyzed by performing an analysis of variance (ANOVA) and Tukey’s test using computer-aided software DSAASTAT.

## Results

### Isolation and identification of leaf spot pathogen

**Figure 2:**
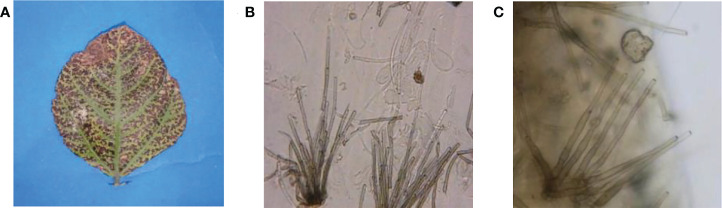
Symptoms caused by *C. canescens* on mungbean leaves **(A)** and microscopic characters of C. canescens observed at 40X magnification **(B, C)**.

Fungal leaf spot pathogen was isolated and purified from infected leaves of *V. radiata* collected during field surveys (**Figure 2A**). The isolated fungal pathogen was preliminary identified as *Cercospora canescens* based on morphological observations. Spores were cylindrical, hyaline, straight to slightly curved, 2-12 septate, 10-300 X 1.3-5.0 micrometer in size (**Figure 2B, C)**. Identification based on morphological features was further confirmed by the amplification and sequencing of the ITS region. Homology analysis showed >99% similarity with the previously submitted *C. canescens* isolates under the accession numbers (MN795697.1), (MN795657.1) and (MN795694.1) (**Supplemental Figure 3**).

### Confirmation of pathogenicity

A detached leaf assay was performed to confirm the pathogenicity of *C. canescens* isolates. Brown spots appeared after four days of inoculation (**Supplemental Figure 1**). The necrosis started rapidly after the 4^th^ day (**Supplemental Figure 2**). Re-isolations and morphological characterizations of the pathogen from symptomatic leaves confirmed the pathogen identity and virulence.

### Characterization of green synthesized ZnO NPs

**Figure 3:**
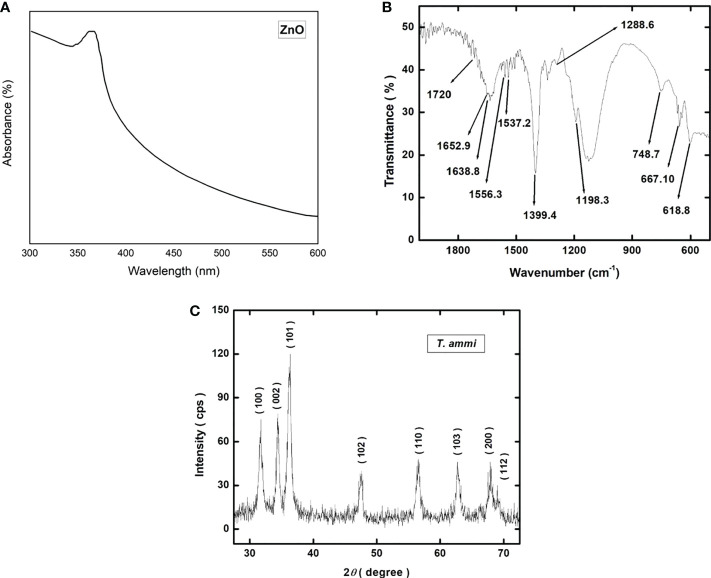
**(A)** UV-vis spectrophotometric plot showing the absorbance spectra of green synthesized ZnO NPs; **(B)** Graphical representation of Fourier-transform infrared spectroscopy (FTIR) spectrum of green synthesized ZnO NPs; **(C)** X-ray diffraction (XRD) pattern of green synthesized ZnO NPs.

The UV-vis absorption spectra of the green synthesized zinc oxide nanoparticles are shown in **Figure 3A**. The main absorption bands of zinc oxide nanoparticles were found at 338 nm (**Figure 3A**). The absorption bands obtained from FTIR spectra of ZnO nanoparticles synthesized using are shown in **Figure 3B**. The spectra were recorded over the range of 4000–450 cm^−1^ using IR an attenuated total reflectance (ATR) assembly. The absorption bands were observed at 618.8, 667.10, 748.7, 1198.3, 1288.6, 1399.4, 1537.2, 1556.3, 1638.8, 1652.9, and 1720 cm^−1^.

The X-ray diffraction pattern of the ZnO nanoparticles is shown in **Figure 3C**. The nanoparticles are polycrystalline showing strong diffraction peaks at the 2*θ* values of 31.713°, 34.401°, 36.216°, 47.533°, 56.556°, 62.796°, 67.938°, and 68.984° corresponding to the crystallographic planes of (100), (002), (101), (102), (110), (103), (112) and (201) respectively. All the diffraction peaks correspond to the hexagonal wurtzite structure of ZnO nanoparticles and match well with the JCPDS card number 00-036-1451 (Padalia and Chanda, 2017). No peak other than ZnO is detected indicating the absence of any second phase particles. The values of crystallite size are given in **Supplemental Table 2**.

**Figure 4:**
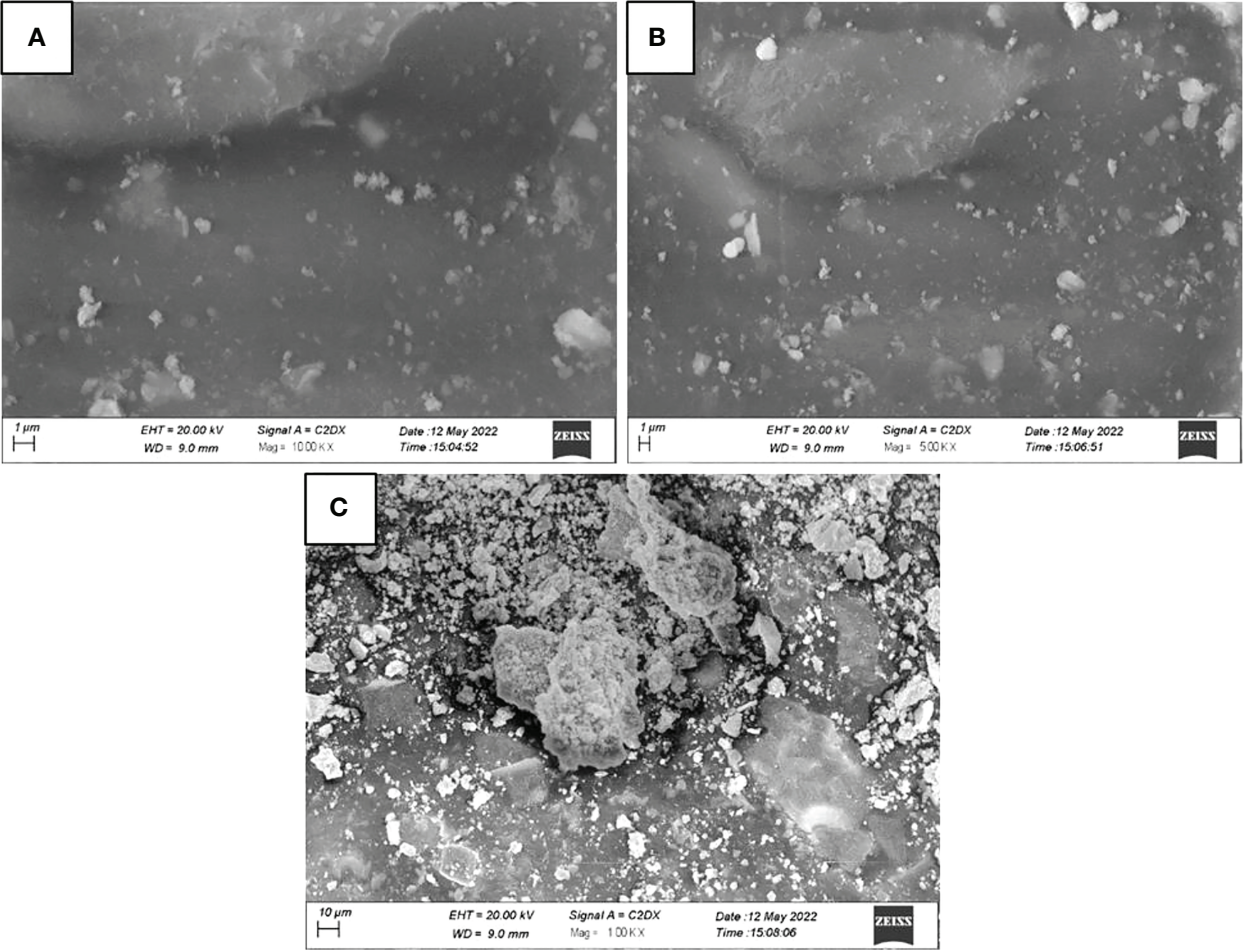
Scanning Electron micrograph of green synthesized ZnO NPs. **(A)** 10kX; **(B)** 5kX; **(C)** 1kX.

High-resolution scanning electron microscopy (HR-SEM) was performed to study the structure, size and shape of zinc oxide nanoparticles (**Figure 4**). Zinc oxide nanoparticles surface was seen as an agglomerated morphology with a cubic shape, with an average size of 15 to 20 nm (**Figure 3C**).

### Antifungal activity of green synthesized zinc oxide nanoparticles

**Figure 5:**
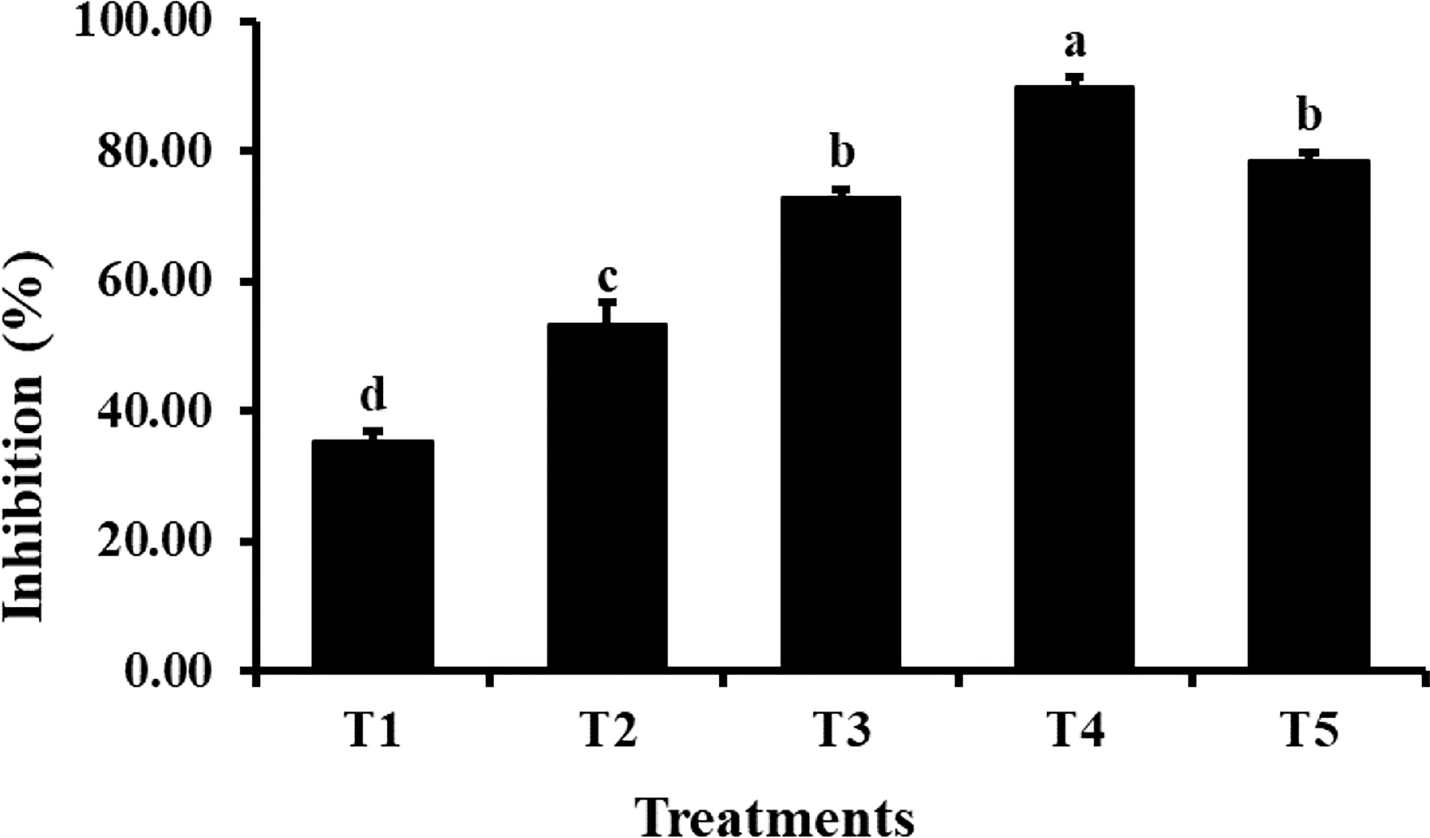
Antifungal activity of different concentrations of ZnO NPs. Vertical bars represent standard error. Different letters indicate a significant difference by Tukey multiple range tests at *p*< *0.05*. Details of treatments can be seen in **Table 1**.

**Figure 6:**
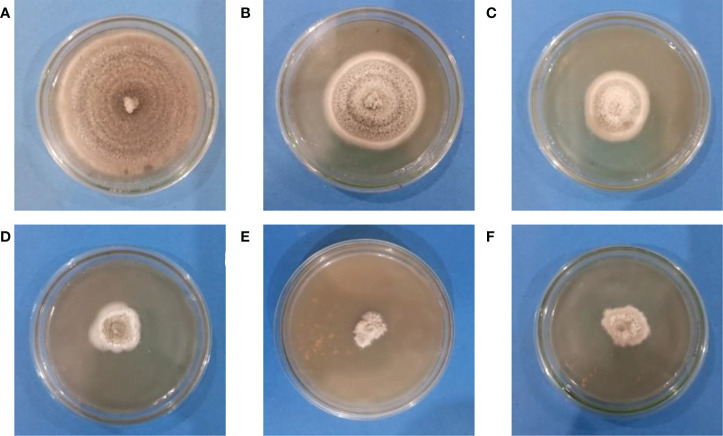
Antifungal activity of Zinc Oxide nanoparticles against *C. canescens* at different concentrations of ZO nanoparticles. **(A)**= 0 ppm; **(B)**= 300 ppm; **(C)**= 600 ppm; **(D)**= 900 ppm; **(E)**= 1200 ppm; **(F)**= 1500 ppm.

The agar dilution method was used to check the antifungal properties of ZnO NPs against *C. canescens*. The radial growth of fungal pathogen was inhibition ranging from 35.44 to 89.85% at all test concentrations (**Figure 5**). Maximum inhibitory activity of 89.85 and 78.65% was observed when NPs were applied at a concentration of 1200 and 1500 ppm, respectively (**Figure 6**). The inhibition activity of green synthesized ZnO NPs was even higher as compared to the fungicides used as a negative control in this experiment (**Figure 6**).

### Potential of ZnO NPs to suppress Cercospora leaf spot disease

**Figure 7:**
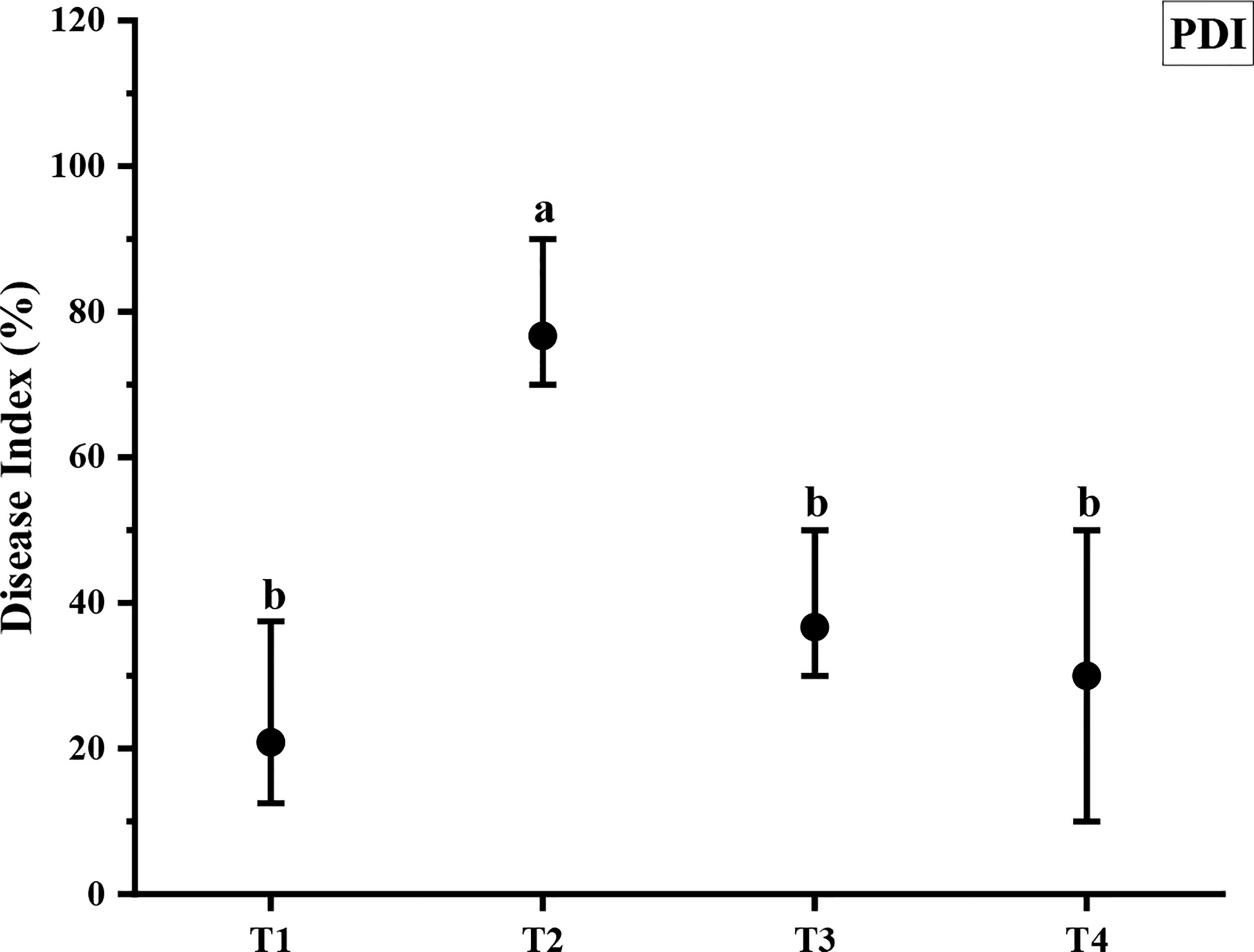
Antifungal efficacy of different concentrations of ZnO NPs against *C. canescens*. Vertical bars represent standard error. Different letters indicate significant differences among different treatments as governed by Tukey multiple range tests at *p*< *0.05*. T1= Negative control; T2= Positive control; T3= ZnO NPs applied ta 900ppm; T4= ZnO NPs applied at 1200ppm.

ZnO NPs significantly reduced the Cercospora leaf spot disease index in Mungbean plants (**Figure 7**). Out of different concentrations used in the greenhouse, ZnO NPs applied at 1200 ppm reduced disease index up to 60.87% compared with the pathogen control (**Figure 7**). However, ZnO NPs applied at 900 ppm reduced disease index up to 52.17% as compared to pathogen control (**Figure 7**).

### Effect of ZnO NPs on agronomic traits of mungbean plants

**Figure 8:**
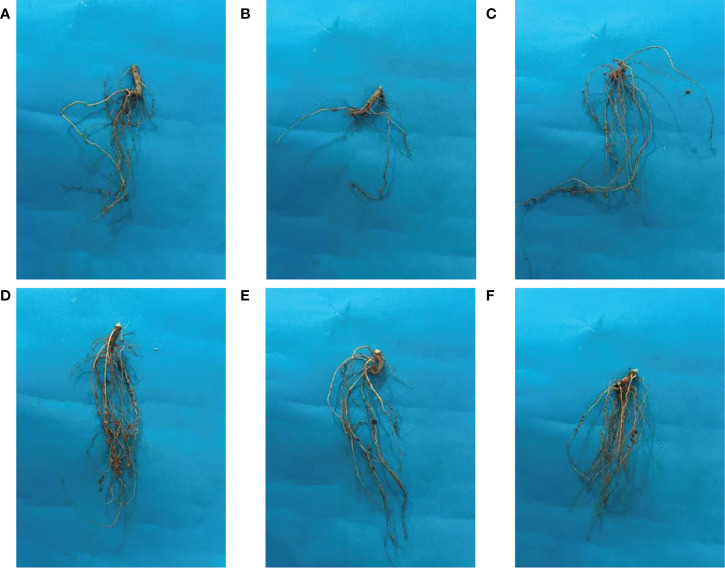
Effect of ZnO NPs on the root morphology of mungbean plants. **(A)** Negative control; **(B)** Positive control; **(C)** ZnO NPs applied at 900ppm; **(D)** ZnO NPs applied at 1200ppm; **(E)**= ZnO NPs applied at 900ppm + *C. canescens*; **(F)** ZnO NPs applied at 1200ppm + *C. canescens*.

The beneficial effect of ZnO NPs on some agronomic traits of Mungbean plants was also examined under greenhouse conditions. Application of ZnO NPs significantly increased growth and yield aspects of mungbean plants both in the presence and absence of fungal pathogens compared with the respective control plants (**Table 2**). Plant growth-related parameters such as shoot length (36%), root length (44.02%), shoot fresh weight (32.82%), root fresh weight (30.36%), shoot dry weight (33.83%), root dry weight (29.6%), the number of pods (41.3%) were significantly increased in plants receiving ZnO NPs (1200 ppm) as compared to non-treated control plants (**Figure 8**). Similarly, phytochemical contents i.e. total chlorophyll (43.02%) and carotenoid contents (14.93%) were significantly increased in ZnO NPs (1200 ppm) in treated plants compared to non-treated control plants (**Table 3**).

**Table 2:** *In-planta* effect of different concentrations of ZnO NPs plant growth parameters.

Treatments	Shoot Length(cm)	Root Length(cm)	Number of Leaves	Number of Pods	Shoot Fresh Weight(g)	Shoot Dry Weight(g)	Root Fresh Weight(g)	Root Dry Weight(g)	Pod Size(cm)	Root Nodules
**T1**	33.32 ± 0.52^c^	15.98 ± 0.56^ab^	32.33 ± 1.37^ab^	15.67 ± 0.52^b^	12.13 ± 0.58^ab^	4.14 ± 0.27^ab^	1.53 ± 0.09^bc^	0.52 ± 0.04^ab^	7.38 ± 0.11^bc^	11.33 ± 0.33^c^
**T2**	20.66 ± 0.87^e^	8.64 ± 0.26^d^	18.67 ± 1.37^d^	9.00 ± 0.89^d^	12.13 ± 0.13^c^	2.70 ± 0.12^c^	1.04 ± 0.05^d^	0.34 ± 0.03^c^	4.77 ± 0.20^d^	6.33 ± 0.67^d^
**T3**	31.15 ± 0.48^d^	14.03 ± 0.51^c^	28.67 ± 1.03^c^	13.33 ± 0.52^c^	11.30 ± 0.58^b^	3.68 ± 0.13^b^	1.41 ± 0.03^c^	0.45 ± 0.04^b^	7.24 ± 0.05^c^	12.33 ± 0.33^c^
**T4**	32.25 ± 0.29^cd^	15.44 ± 0.65^b^	30.67 ± 1.03^bc^	15.33 ± 0.52^b^	12.29 ± 0.48^ab^	4.07 ± 0.15^ab^	1.49 ± 0.01^bc^	0.48 ± 0.02^b^	8.06 ± 0.24^b^	14.67 ± 0.67^ab^
**T5**	34.87 ± 0.47^b^	15.72 ± 0.19^ab^	32.67 ± 1.03^ab^	16.33 ± 0.52^ab^	11.95 ± 0.19^ab^	3.93 ± 0.12^b^	1.63 ± 0.05^b^	0.54 ± 0.03^ab^	7.86 ± 0.13^bc^	13.33 ± 0.33^bc^
**T6**	37.10 ± 0.17^a^	16.98 ± 0.48^a^	34.67 ± 1.03^a^	17.67 ± 0.52^a^	12.96 ± 0.28^a^	4.46 ± 0.22^a^	1.75 ± 0.02^a^	0.58 ± 0.03^a^	8.99 ± 0.19^a^	15.67 ± 0.33^a^

Data are presented as mean values ± Standard error. Different letters indicate significant differences among treatments as governed by Tukey multiple range tests at p< 0.05. Details of treatments can be seen in **Table 1**.

**Table 3:** *In-planta* effect of different concentrations of ZnO NPs on total chlorophyll and carotenoid contents.

Treatments	Chl. A(µg/ml)	Chl. B(µg/ml)	Total Chl.(µg/ml)	Carotenoids(µg/g)
**T1**	0.32615 ± 0.0027^d^	0.23506 ± 0.0026^e^	0.81341 ± 0.006^b^	14.3779 ± 0.0638^bc^
**T2**	0.44563 ± 0.0041^b^	0.36778 ± 0.0018^bc^	0.56121 ± 0.005^e^	13.3658 ± 0.0586^e^
**T3**	0.58085 ± 0.0108^a^	0.37871 ± 0.0041^b^	0.95956 ± 0.009^a^	14.7752 ± 0.1603^b^
**T4**	0.59142 ± 0.0033^a^	0.39349 ± 0.0006^a^	0.98491 ± 0.004^a^	15.7123 ± 0.2132^a^
**T5**	0.34506 ± 0.0025^cd^	0.31536 ± 0.0036^d^	0.66042 ± 0.001^d^	13.7804 ± 0.0920^de^
**T6**	0.36326 ± 0.0059^c^	0.3582 ± 0.0054^c^	0.72146 ± 0.011^c^	14.2447 ± 0.0301^cd^

Data are presented as mean values ± Standard error. Different letters indicate significant differences among treatments as governed by Tukey multiple range tests at p< 0.05. Details of treatments can be seen in **Table 1**.

**Figure 9:**
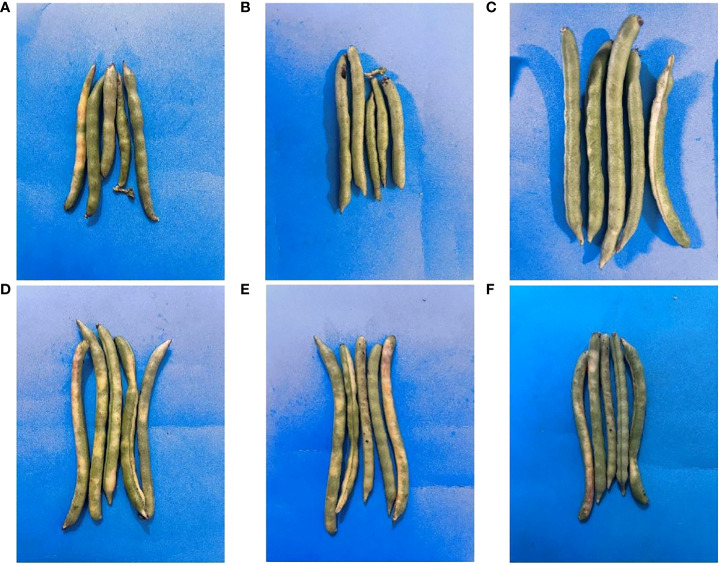
Effect of ZnO NPs on the pods of mungbean. **(A)**= Negative control; **(B)**= Positive control; **(C)**= ZnO NPs *applied* at 900ppm; **(D)**= ZnO NPs *applied* at 1200ppm; **(E)**= ZnO NPs *applied* at 900ppm+ *C. canescens*; **(F)**= ZnO NPs *applied* at 1200ppm + *C. canescens*.

In terms of the yield-related parameters, data indicated significant treatment effects (**Table 2**). However, the treatment effect was more evident when ZnO NPs were applied at higher concentrations (1200 ppm). Plants treated 1200 ppm with ZnO NPs produced significantly greater pod numbers per plant (41.3%), and pod size (40.86%) compared with the non-treated control plants (**Figure 9**).

A further treatment effect was apparent on root nodulation. The plants exposed to ZnO NPs (1200 ppm) achieved the highest number of root nodulations compared to the rest of the treatments. The respective number of root nodulation treated with 1200 ppm ZnO NPs (50%) and 900 ppm ZnO NPs (41.67%) were all significantly higher compared to the non-treated control plants. Similar findings were observed regarding the beneficial effect of ZnO NPs on agronomic traits of Mungbean plants in the presence of leaf spot pathogen, hence they are not discussed.

## Discussion

This work has illustrated the successful isolation, characterization, and environment-friendly management of Cercospora leaf spot fungus. The isolated pathogen, *C. canescens*, is recognized as the most harmful pathogen of mung bean in Pakistan (Abbas et al., 2020). Scientists have been greatly concerned with this disease, and several strategies have been used for its control. For the eco-friendly synthesis of ZnO NPs, *T. ammi* seed extract with the best antifungal activity was used. The presence of alkaloids and flavonoids may be a source of antifungal potency of *T. ammi*, which are known to be biologically active chemicals against fungus and bacteria (Ali et al., 2022). The bioactive polyphenols in seed extracts are reported to disrupt the life cycle of fungi by attaching to their protein molecules, changing the production of structural components, weakening or dissolving the cell membrane permeability barrier, and changing the physiological state of the cell (Rongai et al., 2015). Due to the presence of several antifungal compounds in the seed extract of *T. ammi*, previous studies have demonstrated the antifungal potential of this plant against various fungal pathogens (Arif et al., 2021). Observed FTIR spectra of the synthesized ZnO NPs confirmed the presence of thymol, a well-known phenolic antibacterial molecule (Dheer et al., 2019). Additionally, copper, silver, nickel, and magnesium green nanoparticles have already been synthesized using *T. ammi* extract (Jagana et al., 2017).

In this work, seed extract was used to conduct the green synthesis of ZnO NPs. According to the previous studies, bioactive substances found in *T. ammi* seed extracts may interact with functional groups of metal nanoparticles which function as reducing agents, to adsorb on the surfaces of these particles. Compounds including amino acids, polyphenols, nitrogen bases, and reducing sugars are abundant in plants. During the synthesis of magnetite nanoparticles, these compounds act as stabilizing and reducing agents (López and Antuch, 2020). Due to their stability and small size, the produced green ZnO NPs in this work demonstrated increased antifungal activity. Zinc ions inside bacterial cells were also engaged in the disruption of metabolic activities. Zinc ions released later may be attached to DNA molecules and caused disorder of the helical structure by cross linking inside and between the nucleic acid strands (Chikkanna et al., 2018). Smaller size increases the dispersion and penetration of the intracellular matrix and interferes with intracellular absorption of Ca^2+^ and induces cell damage (Srihasam et al., 2020). Damage to the cell membrane and internal organelles occurs as a result of the attachment of NPs to the microbial intracellular cell membrane (Basak et al., 2014). The production of reactive oxygen species (ROS), such as hydrogen peroxide (H_2_O_2_) and hydroxyl radicals (OH¯), is another mechanism for antimicrobial activity. ROS play a role in various mechanisms, including cell wall damage due to ZnO-localized interaction, increased membrane permeability, internalization of nanoparticles due to loss of proton motive force, and uptake of toxic soluble zinc ions (Bayat et al., 2019). Up-regulation of PR1 and PR2 genes were observed in *Nicotiana benthamiana* treated with ZnO and SiO2 NPs (Cai et al., 2019).

ZnO NPs have great potential to enhance plant growth-related parameters and physiological processes in plants. The results of current experiment indicated that 900 and 1200 ppm of green synthesized nanoparticles significantly enhanced all growth parameters including plant height, number of leaves, and total dry matter content in comparison to other concentrations. These results were in support of another experiment in which foliar application of nano chelate zinc increased 94% of grain yield in the maize crop (Mosanna and Behrozyar, 2015). Significant increase in the above growth parameters might be associated with a better nutritional environment in the root zone for the growth and development of plants as well as in the plant system under the influence of improved availability of different nutrients due to the application of ZnO NPs. It is a fact that Zinc is an essential micro-nutrient required for plant nutrition. ZnO NPs showed greater positive effects as compared to ZnO bulk treatments. We concluded that ZnO NPs into soluble ions in solution (water or soil solutions) causing discharge of the micronutrient (Zn). The amount and speed of dissolution are considerably higher than bulk fertilizers due to the reduced particle size and greater specific surface area of ZnO NPs. This solubility of ZnO NPs helped the growth and physiological parameters of the plant.

A significant increase in the physiological processes of the plants was observed where the chlorophyll and carotenoid contents increased with the increase in the concentration of ZnO NPs. These results depicted that the nanoparticle application greatly suppressed the pathogen and increased plant growth. Similar results were reported by Prasad et al. (2012) which resulted in increased seed germination, along with enhanced seedling vigor in peanut seeds and increased leaf chlorophyll contents when treated with 1000 mg/kg of ZnO NPs. In addition, Dimkpa et al. (2020) showed the beneficial effect of ZnO NPs on the plant height, shoot biomass, chlorophyll, grain yield, and uptake of nutrients in wheat crops, with 40% field moisture capacity. Studies carried out in seeds and seedlings of ragi (finger millet) showed the effects of ZnO-NPs as an improvement in the vigor of 45.54% with treatments of 1000 ppm was reported, while at 1500 ppm a significant reduction of this parameter was observed (Rameshraddy et al., 2017). Similar results were found in *Capsicum annuum* L. seeds exposed to 0.75 g of ZnO-NPs, that increased the vigor index significantly (67%) (Afrayeem and Chaurasia, 2017). Several studies have demonstrated that applying ZnO NPs to soils can have positive effects on plants at the physiological, morphological, biochemical, and molecular levels. For example, ZnO NPs enhanced photosynthesis and biomass in lettuce (Xu et al., 2018). Similar studies with the increase in the growth parameters of the plant were observed in a dose-dependent manner (Awasthi et al., 2017). Similar physiological results have been observed previously by (Leopold et al., 2022). The study reported a 23% increase in the chlorophyll contents at concentrations of 1000ppm. Green synthesized ZnO NPs were given as the nutrient source for the growth of the sesame plant with different concentrations (1, 3, 5, 7, 9 mg/ml). The concentration of 5 mg/ml of ZnO NPs revealed significant (*p*< 0.05) growth in root and shoot development of the plant when compared to the control (Arumugam et al., 2021). Similar concentration of ZnO nanoparticles increased the total chlorophyll in Helianthus annuus (Rajiv et al., 2018). Some former studies also specified that soil and foliar treatment of TiO2, ZnO and CuO nanoparticles enhanced lycopene content in tomato plants (Pérez-Labrada et al., 2019; Sharifan et al., 2022).

The current study includes the use of ZnO NPs to control disease and improve growth attributes and physiological parameters. The concentrations of the used ZnO NPs suspensions were 0, 300, 600, 900, 1200, and 1500 ppm. In the case of the NPs used, only the use of the 1200 ppm ZnO NPs resulted in a decrease in the plant disease index (PDI). Good antimicrobial efficacy in the lab and under field conditions was observed by the green synthesized ZnO NPs against the Cercospora leaf spot of mung bean. These results determine the promising ability of these synthesized nanoparticles in the field of biology and especially agricultural plant pathology.

## Data availability statement

The original contributions presented in the study are included in the article/**Supplementary Material**. Further inquiries can be directed to the corresponding authors.

## Author contributions

Conceived and designed the total experiments: Z-E-HA, TA. Performed the experiments: HR, FM, MA. Analyzed data: MA, UB, GL. Wrote the paper: Z-E-HA, TA. Funding Procurement: GL. All authors contributed to the article and approved the submitted version.

## Funding

This study was financially supported by the Guangdong Province Key Areas Research and Development Plan (Project No: 2022B0202110003); Guangdong Province Special Fund for Modern Agriculture Industry Technology Innovation Teams (Project No: 2022KJ122 and 2023KJ122) and China Young Scientist Talent Program (Project No: QN2022030024L).

## Conflict of interest

The authors declare that the research was conducted in the absence of any commercial or financial relationships that could be construed as a potential conflict of interest.

## Publisher’s note

All claims expressed in this article are solely those of the authors and do not necessarily represent those of their affiliated organizations, or those of the publisher, the editors and the reviewers. Any product that may be evaluated in this article, or claim that may be made by its manufacturer, is not guaranteed or endorsed by the publisher.
