# Supplementary material for: Bio-fabrication of Zinc Oxide nanoparticles to rescue Mung Bean against Cercospora leaf spot disease
Source: Front Plant Sci. 2022 Nov 29;13:1052984. doi: 10.3389/fpls.2022.1052984 (PMC9745094; doi:10.3389/fpls.2022.1052984)
Supplement: Supplementary file 1 [file DataSheet_1.docx]

**Supplemental Table 1.** The disease rating scale used for the estimation of Plant Disease Index (PDI).

| **Severity rating** | **Symptom Description** | **Response** |
| --- | --- | --- |
| 0 | No visible symptoms on plants | Highly Resistant |
| 1 | 1-20% foliage or pod area affected with small pinhead lesions | Resistant |
| 3 | 21-40% foliage or pod area affected with small round brown spots | Moderately Resistant |
| 5 | 41-60% foliage or pod area affected with large spots | Moderately Susceptible |
| 7 | 61-75% foliage or pod area affected with bigger coalescing spots | Susceptible |
| 9 | 76-100% foliage or pod area affected with bigger coalescing spots | Highly Susceptible |

**Supplemental Table 2:** Details of crystallite size *D* using Scherrer formula.

| **2**$\boldsymbol{\theta}$**^o^** | $\boldsymbol{\theta}$**^o^** | **FWHM** | ***D* (nm)** |
| --- | --- | --- | --- |
| 31.713 | 15.8565 | 0.0024979 | 60.26534 |
| 34.401 | 17.2005 | 0.0033115 | 45.77762 |
| 36.216 | 18.108 | 0.0042071 | 36.21452 |
| 47.533 | 23.7665 | 0.0074587 | 21.21478 |
| 56.556 | 28.278 | 0.0057192 | 28.7511 |
| 62.796 | 31.398 | 0.0040743 | 41.63996 |
| 67.938 | 33.969 | 0.0023123 | 75.51999 |
| 68.984 | 34.492 | 0.0030773 | 57.09726 |

**Supplemental Figure 1.** Disease symptoms caused by C. canescens on detached mungbean leaves. A= 1st day post inoculation. B= 8^th^ day post inoculation.


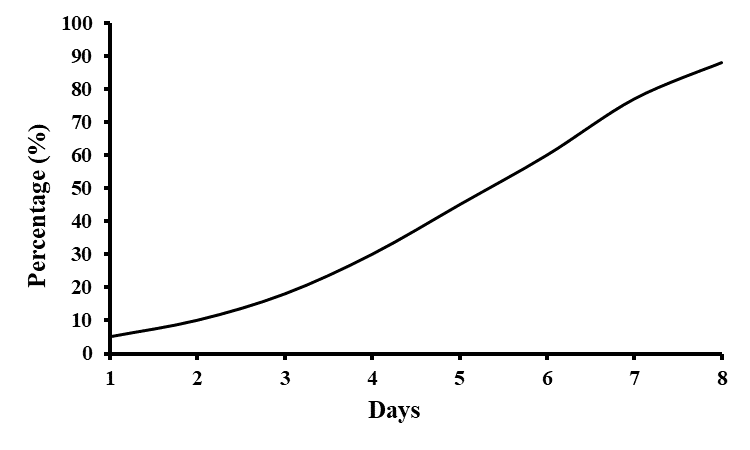


**Supplemental Figure 2.** Cercospora leaf spot disease progression curve up till 8^th^ day post inoculation.


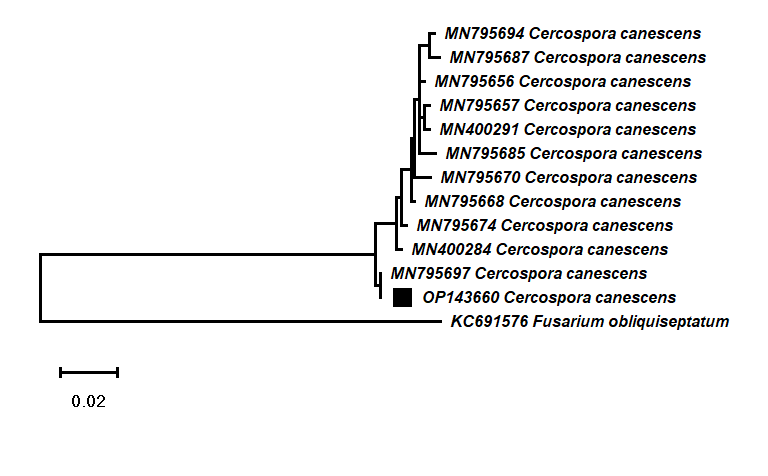


**Supplemental Figure 3.** The maximum likelihood tree of *C. canescens* isolates (OP143660) based on the ITS sequences.


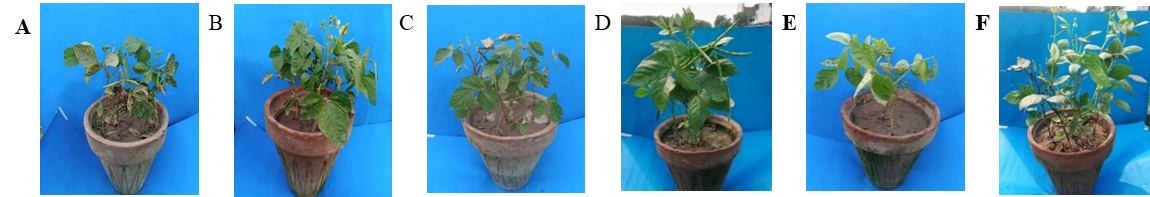


**Supplemental Figure 4.** In-vivo antifungal activity of ZnO NPs against C. canescens. A= Negative control; B= Positive control; C= 900ppm; D= 1200ppm; E= 900ppm + C. canescens; F= 1200ppm + C. canescens.


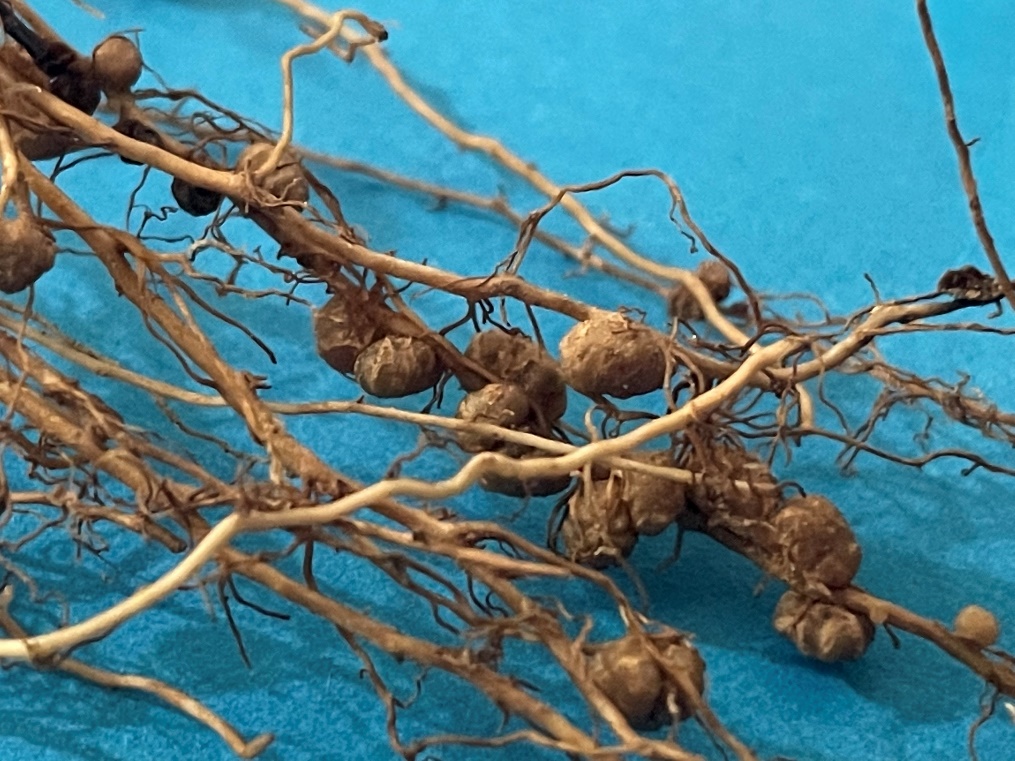


**Supplemental figure 5.** Presence of root nodules in mungbean plants treated with ZnO NPs.
